# An Electrochemical Ti_3_C_2_T_x_ Aptasensor for Sensitive and Label-Free Detection of Marine Biological Toxins

**DOI:** 10.3390/s21144938

**Published:** 2021-07-20

**Authors:** Najeeb Ullah, Wei Chen, Beenish Noureen, Yulan Tian, Liping Du, Chunsheng Wu, Jie Ma

**Affiliations:** 1 Department of Biochemistry and Molecular Biology, School of Basic Medical Sciences, Xi’an Jiaotong University Health Science Center, Xi’an 710061, China; Ullahnajeeb@stu.xjtu.edu.cn; 2 Institute of Medical Engineering, Department of Biophysics, School of Basic Medical Science, Xi’an Jiaotong University Health Science Center, Xi’an 710061, China; weiwcchen@xjtu.edu.cn (W.C.); Beenishktk@stu.xjtu.edu.cn (B.N.); cnyulantian@stu.xjtu.edu.cn (Y.T.); duliping@xjtu.edu.cn (L.D.); 3 Medical Research Center, Xi’an No.3 Hospital, Xi’an 710018, China

**Keywords:** Ti_3_C_2_T_x_, aptamer, saxitoxin, marine toxins, electrolyte-insulator semiconductor

## Abstract

Saxitoxin (STX) belongs to the family of marine biological toxins, which are major contaminants in seafood. The reference methods for STX detection are mouse bioassay and chromatographic analysis, which are time-consuming, high costs, and requirement of sophisticated operation. Therefore, the development of alternative methods for STX analysis is urgent. Electrochemical analysis is a fast, low-cost, and sensitive method for biomolecules analysis. Thus, in this study, an electrolyte-insulator-semiconductor (EIS) sensor based on aptamer-modified two-dimensional layered Ti_3_C_2_T_x_ nanosheets was developed for STX detection. The high surface area and rich functional groups of MXene benefited the modification of aptamer, which had specific interactions with STX. Capacitance-voltage (*C-V*) and constant-capacitance (ConCap) measurement results indicated that the aptasensor was able to detect STX with high sensitivity and good specificity. The detection range was 1.0 nM to 200 nM and detection limit was as low as 0.03 nM. Moreover, the aptasensor was found to have a good selectivity and two-week stability. The mussel tissue extraction test suggested the potential application of this biosensor in detecting STX in real samples. This method provides a convenient approach for low-cost, rapid, and label-free detection of marine biological toxins.

## 1. Introduction

Paralytic shellfish poisoning (PSP) toxins are shellfish toxins synthesized by microscopic dinoflagellates [1,2]. The microorganisms englobed by shellfish accumulate toxins in their body and spread through the food chain or water contamination. Saxitoxin (STX) belongs to the PSP toxins and leads to numbness of organs, breathing difficulties, and death in humans [3,4,5]. Besides, fatal illnesses have been known to occurs in some cases without effective antidotes [6]. For drinking water, 3 ng mL^−1^ of STX (toxicity equivalents) is the guidance value implemented in Australia, Brazil, and New Zealand [7]. Thus, it is important to detect STX in seafood or drinking water. Traditional approaches for STX analysis include mouse bioassay [8], enzyme-linked immunosorbent assay (ELISA) [9], cell assay [10], high-performance liquid chromatography (HPLC) [11], and liquid chromatography-tandem mass spectrometry (LC-MS) [12]. Although respective advantages in sensitivity and specificity have been achieved by these methods, there are some challenges: for example, HPLC and LCMS need sophisticated equipment and complex pre-treatments [13,14], and ELISA requires tedious lab work [15]. To address these challenges, electrochemical analysis with functional nanomaterials could be a potential solution [16]. Bioactive materials, such as nucleic acid and antigen-antibody combined with electrochemical detectors, were developed for biomolecule detection with convenient operation, high sensitivity, and good specificity [17]. Aptamer, a single-standard oligonucleotide is an ideal candidate. Aptamer is prepared by a technique called systematic evolution of ligand exponential enrichment (SELEX) and has specific binding to the target molecules [18]. Typically, aptamer has decisive advantages compared to antibodies, such as low cost, high selectivity, high stability, and easy synthesis and functionalization [19]. Thus, aptamer has been widely used in many fields, including biomolecule detection [20,21,22] and cell analysis [23].

Nanomaterials with high surface area, unique morphology and structure, and functional groups have been widely applied in biosensors as sensitive elements or signal enhancers to improve electrical conductivity, selectivity, and sensitivity [24,25]. There are four types of nanomaterials: zero-dimension, one-dimension, two-dimensions, and three-dimensions. MXene belongs to two-dimensional nanomaterial, which has attracted much attention due to its graphene-like structure and morphology, rich functional groups, and excellent electrical conductivity [26]. M_n+1_X_n_T_x_ represents the composition of MXene, which is usually derived by etching A atoms (A is ⅢA or ⅣA element) from the MAX phase (where M represents a transition metal, X represents carbon or nitrogen, and T_x_ represents surface terminal groups like -O, -F, and -OH) [27]. MXene has been reported in various fields, ranging from battery material, gas separation, environmental control, to nanomedicine [28,29,30,31]. The hydrogen bonds, electrostatic interactions, and van der Waals forces between oxygen or hydroxyl groups of MXenes and biomolecules result in the applications of MXene as a sensitive part or matrix in biosensors. For instance, based on the large surface area, good conductivity, and catalytic properties, aptamer covalently modified MXene nanosheets used as nanoprobes exhibited high sensitivity to exosomes in an electrogenerated chemiluminescence (ECL) sensor [32]. The biosensor exhibited a low detection limit of 125 particles μL^−1^, 100 times lower than that of the conventional ELISA method. Similarly, gold nanoparticles-decorated MXene with aptamer modification was also reported as an exosome biosensor through the ECL method [33]. The detection limit was 30 particles μL^−1^ for exosomes derived from HeLa cell lines, lower than that of the ELISA method. The high sensitivity was ascribed to the synergistic effects of large surface area, excellent conductivity, and catalytic effects of the composite. Recently, a comprehensive review by Ding et al. discussed the development of MXenes applications in tactile sensors, memristors, and artificial tactile perception systems [34]. The synthesis, properties, and applications of MXene were summarized and discussed systemically with a profound challenge and perspective.

In this work, aptamer modified MXene was utilized to develop a novel aptasensor for sensitive and label-free detection of a marine biological toxin, STX. MXene was able to improve the efficiency of aptamer binding and immobilization due to its high surface area and rich functional groups. An electrolyte-insulator-semiconductor (EIS) structure was used as a transducer for STX detection. Capacitance-voltage (*C-V*) and constant-capacitance (ConCap) measurement were carried out to monitor the changes in sensor capacitance, resulting from the specific interactions of aptamer and STX. In principle, the conformational changes of aptamer lead to charge changes in the sensor surface, which resulted in changes in sensor capacitances. Thus, the sensor was able to detect STX by monitoring changes in its capacitances.

## 2. Materials and Methods

### 2.1. MXene Synthesis

Multilayer Ti_3_C_2_T_x_ was prepared in reference to a previous report [35]. Briefly, 100 mg of Ti_3_C_2_T_x_ (lab synthesized) was added to 20 mL tetrapropylammonium hydroxide (TPAOH, Sinopharm Chemical Reagent Co., Ltd., Shanghai, China) solution (5 wt.%) and stirred for 30 min. Then the mixture was placed in an oven under 140 °C for 24 h. Precipitation of the mixture was removed via centrifuging at 3500 rpm for 30 min. Then, deionized water was used to wash the collected supernatant several times. The collected sample was placed in an ultrasonic bath (200 W) for 2 h. Finally, the product was dried at 50 °C in a vacuum oven overnight.

### 2.2. Sensor Preparation and Functionalization

The sensor was prepared according to [36] with modifications. An EIS structure (Au/n-Si/SiO_2_) was grown on a silicon wafer (*n*-type, <100>, 10–15 Ωcm) (Schematic 1). First, a layer of SiO_2_ (30 nm) was prepared on the silicon wafer by dry oxidization to serve as an insulation layer. Then, hydrofluoric acid (HF, Sinopharm Chemical Reagent Co., Ltd., Shanghai, China) was employed to etch the rear side of the wafer to get rid of the SiO_2_ layer. Next, a layer of gold was deposited on the etched rear side of the wafer. Then, the wafer was ready for further experiments after cutting into small pieces with desired sizes and cleaning in an ultrasonic bath with acetone, isopropyl alcohol, ethanol, and deionized water, sequentially.

The aptamer (5′-end amino-modified STX aptamer (5′-GGTAT TGAGG GTCGC ATCCC GTGGA AACAT GTTCA TTGGG CGCAC TCCGC TTTCT GTAGA TGGCT CTAAC TCTCC TCT-3′)) modified Mxene was used to modified the surface of the EIS sensor by a chemical process. Briefly, 0.1 mL of 1% silane coupling agent (Sinopharm Chemical Reagent Co., Ltd., Shanghai, China) in 10 mL ethanol (Sinopharm Chemical Reagent Co., Ltd., Shanghai, China) was mixed with 2 mL of 50 mg/mL MXene and kept overnight at room temperature (RT) under stirring. After centrifuging and washing with ethanol for two times, the precipitation was dispersed in a 10 mL of tetrahydrofuran (THF) (Sinopharm Chemical Reagent Co., Ltd., Shanghai, China) and mixed with 0.5 µL of 100 µM aptamers (Sangon Biotech (Shanghai) Co., Ltd.) and 31 µL of triethanolamine (TEA) (J&K Scientific Ltd., Beijing, China) in an ice bath for 10 min, and then reacted at 50 °C for 24 h with stirring. The collected product was washed with THF and water for several times, and added to the Au/n-Si/SiO_2_ substrate in an oven at 80 °C to dry 30 min. For each test, the sensor surface was incubated with varied concentrations of STX solution (room temperature, 10 mM NaCl, pH 5.45) for 1 h at room temperature before the EIS analysis. To optimize the immobilization, various volumes of the MXene-aptamer (1 mg mL^−1^) were dropped on the surface of the substrate. An optimal MXene-aptamer film should be firm, stable, and electroactive. During the tests, the electrodes soaking 10 mM NaCl solution were kept in 4° C refrigeration.

### 2.3. Measurements

**Scheme 1:**
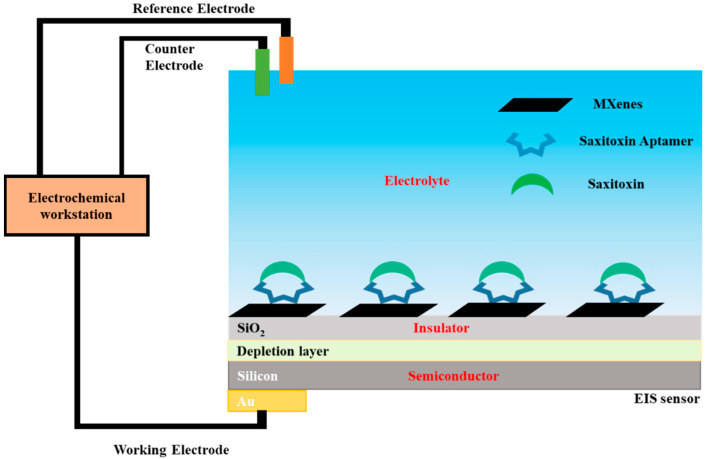
Schematic diagram of the MXene-Aptamer biosensor for STX detection.

The electrochemical measurements were performed on an electrochemical workstation (Zennium, Zahner Elektrik, Bad Staffelstein, Germany). A Pt wire (counter electrode), Ag/AgCl (reference electrode), and EIS sensor (working electrode) formed a 3-electrode electrochemical measurement system (Scheme 1). A low-ionic strength NaCl solution (10 mM, pH 5.45) was used as the measurement solution. For STX detection, a homemade measurement chamber was utilized to fix the EIS sensor. The chamber was fabricated by an EIS substrate (working electrode), silicon rubber (seal ring), and plastic holder (container, a square hole in the bottom) from bottom to top. Capacitance changes were monitored by capacitance-voltage (*C-V*) and constant-capacitance (ConCap) measurements. For *C-V* measurements, a direct current (DC) gate voltage (−0.5 V to +1.5 V, steps of 100 mV) and a small superimposed alternative current (AC) voltage (20 mV, 60 Hz) was employed. For ConCap measurements, the gate voltage with a feedback control circuit was applied under a fixed capacitance determined by *C-V* measurements [33]. To reduce the influence of electromagnetic fields, the measurement chamber was shielded by a Faraday box.

The real sample test had the following steps: the mussel tissue was taken out of the shells and washed with DI water. Then, 2 mL 50% methanol was added to 0.5 g mussel tissue by strong vortexing for 5 min. The supernatant was collected by the centrifugation method. Then the solution was heated to 75 °C for 5 min, followed again by centrifugation. The supernatant was mixed with a given STX to form a real testing sample.

### 2.4. Characterization

The morphologies of Ti_3_AlC_2_ and Ti_3_C_2_T_x_ were investigated by a Hitachi SU-70 scanning electron microscope (SEM). A FEI/Philips Tecnai 12 BioTWIN transmission electron microscope (TEM) was used to observe the morphology of nanosheets at an acceleration voltage of 120 kV. Atomic force microscopy (AFM) was carried out by a SCANASYST-AIR probe with a nominal tip radius of ~2 nm. Thermo Fisher Nicolet 6700 Fourier transform infrared spectroscopy (FTIR) was carried out in the range of 400 to 4000 cm^−1^ at room temperature.

## 3. Results

### 3.1. Characterization of Aptamer Modified MXene

**Figure 1:**
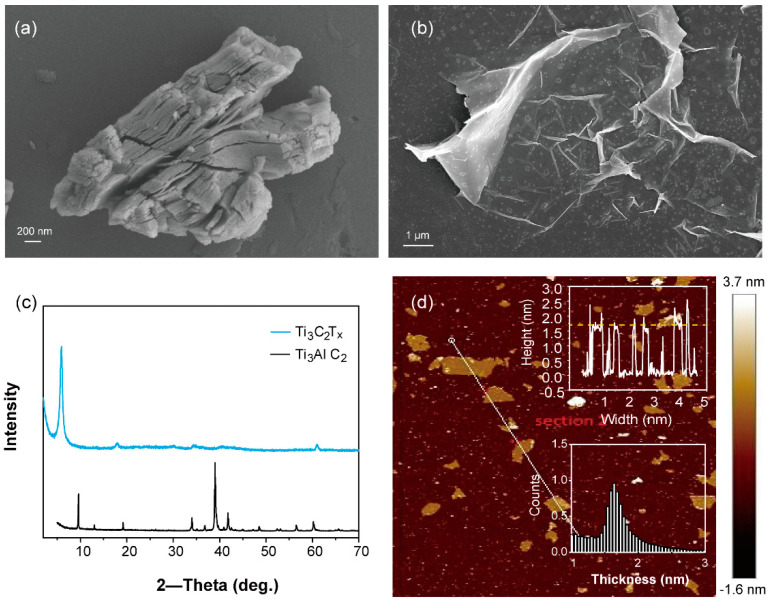
SEM images of (**a**) multilayered Ti_3_C_2_T_x_ before and (**b**) after delamination. (**c**) XRD pattern of Ti_3_AlC_2_ and delaminated Ti_3_C_2_T_x_. (**d**) AFM characterization of delaminated Ti_3_C_2_T_x_.

As shown in Figure 1a, an organ morphology of MXene was an indication of Al atom removal from Ti_3_AlC_2_. When treated by TPAOH and ultrasounication, the samples exhibited graphene-like morphology (Figure 1b). The AFM test indicated that the thickness of the nanosheets was around 1.6 nm (Figure 1d). In the XRD pattern, the (002) peak shifted from 9.50° to 5.84° after etching and delaminating, suggesting an expanded interlayer space (Figure 1c) [35].

**Figure 2:**
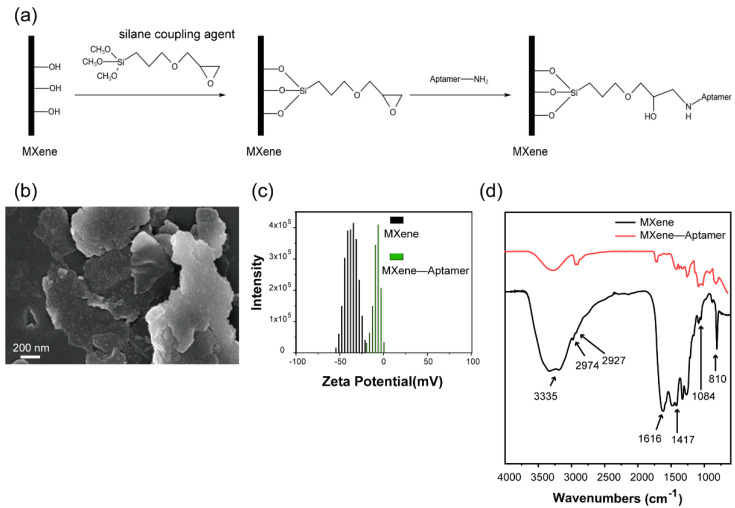
(**a**) Schematics of the preparation of MXene-Aptamer. (**b**) SEM image of MXene-Aptamer. (**c**) Zeta potential of MXene and MXene-Aptamer. (**d**) FITR spectra of MXene and MXene-Aptamer.

The surface of the delaminated Ti_3_C_2_T_x_ was terminated by -OH, -H, and -F groups [37]. Therefore, the aptamer modification was processed, as schematically shown in Figure 2a. The reaction of the hydroxyl groups of MXene with silane coupling agents created glycidyl groups, which further reacted with the amino-terminated aptamer to covalently bond the aptamer to MXene. After the reactions, assembled nanoflakes were observed (Figure 2b) [35]. The existence of the -NH group would balance the negative charge, leading to a decrease in Zeta potential (from −35.5 mV to −6.09 mV) (Figure 2c). The broad peak around 3335 cm^−1^ in the MXene-aptamer was the characteristic peak of –OH (Figure 2d). The N-H peak, a reaction product of glycidyloxypropyl of the silane coupling agent with an amino group of aptamer, was also located there [38]. The peaks at 1084 and 810 cm^−1^ indicated the presence of Si-O-Si [39]. The peaks at 1616 and 1427 cm^−1^ were attributed to N-H and C-N, respectively [40]. These results indicated the successful modification of MXene by the aptamer (named MXene-Aptamer).

### 3.2. Real-Time Monitoring of STX

**Figure 3:**
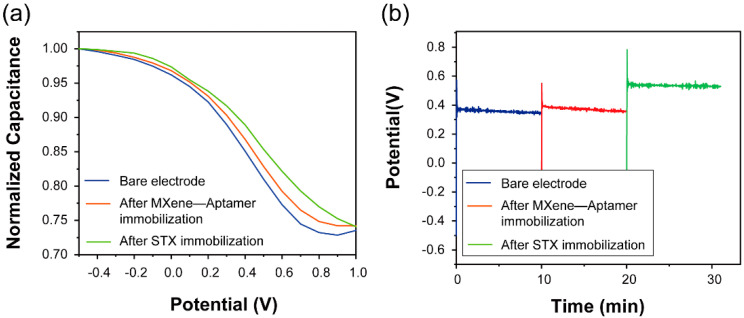
(**a**) *C-V* curves and (**b**) ConCap measurements (working point 2.77 nF) of bare electrodes, MXene-Aptamer immobilization, and 100 nM STX immobilization.

The attachment of STX onto the EIS sensor surface resulted in DNA orientation and changes in surface charge, which lead to the changes in sensor capacitance measured by the shifts in *C-V* curves and potential changes in ConCap mode (Figure 3) [41]. Figure 3a showed the *C-V* curves of the bare electrode, modified with MXene-Aptamer, and STX immobilization, respectively. A typical high-refinery shape with an accumulation region, depletion region, and inversion region was observed. After MXene-Aptamer immobilization, a shift of *C-V* curve towards high voltage direction was observed. Application of STX to the MXene-Aptamer modified sensor led to further shifts of the *C-V* curve to the higher voltage direction of the gate voltage. The specific interactions between STX and aptamer resulted in the conformation changes of aptamer and redistribution of charges on the sensor surface. Therefore, the gate voltages of the *C-V* curve shifted to the higher voltage direction of the gate voltage.

ConCap measurement was employed to realize the real-time monitoring of the potential shifts resulting from surface charge changes. Figure 3b was the dynamic ConCap measurement results after MXene-Aptamer immobilization and STX attachment. A positive potential shift was observed after the MXene-Aptamer immobilization, which was mainly attributed to charge changes originating from the MXene-Aptamer modification on the sensor surface. Similarly, after STX attaching, the conformation changed and charge redistribution led to an additional potential shift to a higher voltage direction.

### 3.3. STX Detection

**Figure 4:**
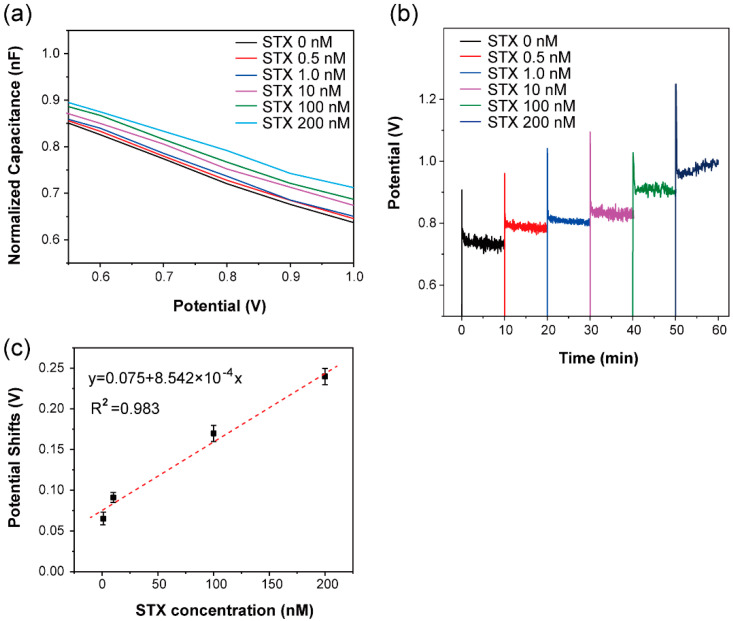
(**a**) *C-V* curves and (**b**) ConCap measurements of the biosensor to STX (from 1.0 nM to 200 nM). (**c**) Statistical results of ConCap measurements; the experiment was repeated three times.

Various concentrations of STX (from 0.5 nM to 200 nM) were used in the STX detection experiments. Figure 4a showed the magnified *C-V* curves in the depletion region of different concentrations of the STX treatment. The curves shifted to the positive direction after STX attachment, as previously. Moreover, larger shifts were found when higher concentrations of STX was added. The ConCap results indicated larger potential shifts along with higher concentration of STX, which were in accordance with the *C-V* tests (Figure 4b). The statistical results of STX to potential shifts indicated a linear response of STX to potential shifts in the range of 1.0 nM to 200 nM. The relationship of potential shift and concentration of STX were described by the equation: *Potential shift* = 0.075 + 8.542 × 10^−4^ *Concentration*. When 0.01 nM and 0.02 nM STX were added separately, a similar potential shift was observed (0.027 V). However, when the concentration of STX increased to 0.03 nM, the potential shift moved to 0.038 V. As the deviation of background (MXene-Aptamer) was 0.006 V, the detection limit of this biosensor was 0.03 nM (S/N = 6.3).

### 3.4. Selectivity and Stability of the Sensor

**Figure 5:**
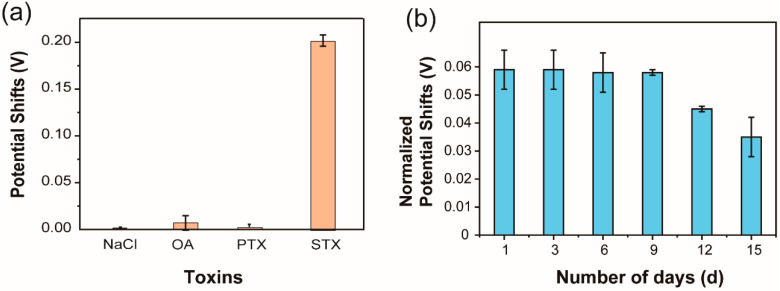
(**a**) Selectivity test of the aptamer sensor to 10 nM NaCl, 1 nM STX, 10 nM PTX, and 10 nM OA. (**b**) Stability test of the MXene-Aptamer sensor to 1 nM STX. All the experiments were repeated three times.

Two other diarrhetic shellfish poisoning (DSP) toxins:pectenotoxins (PTX) and okadaic acid (OA) were selected to verify the selectivity of this aptasensor. The aptasensor responded to STX (1 nM) and the other two toxins (10 nM) were recorded (Figure 5a). Compared with PTX and OA, the responses to STX were significantly higher, indicating good selectivity. An aptasensor with 1 nM STX was stored at 4 °C for 15 days to test the stability of the prepared aptasensor. The biosensor was stable in the first 9 days, then the response gradually decreased (Figure 5b). The longer storage may destroy, oxidize, and peel off the MXene-aptamer film, resulting in the decrease of potential shift.

The reported label-free STX aptasensors are summarized and listed in Table 1 [42,43,44,45,46,47]. Notably, our proposed aptasensor has a relative lower detection limit and moderate detection range. Besides, this method is easy to operate, showing potential applications in environmental pollution monitoring and toxin detection.

The application of this biosensor in a real sample test was performed in mussel tissue extraction. The STX was added to mussel tissue extraction, followed by the same EIS measurement. The rate of recovery (%) was defined as Δ*P_mussel tissue extraction_*/Δ*P_buffer_* (Δ*P* was potential shifts). From Table 2, a slightly increased signal in mussel tissue extraction is seen compared with that of the buffer, suggesting the good stability and recovery of this biosensor for real sample detection. This result indicates that the proposed biosensor could be successfully applied to real sample tests.

## 4. Discussion

Aptamers are able to specifically bind to STX, resulting in conformational change and charge redistribution. In MXene-Aptamer, these two parameters were able to lead to a change in surface charge in MXene and the insulating layer, which was reflected by the capacitance change (Scheme 1). Indicated by Figure 3, the gate voltages of the *C-V* curves shifted to a positive direction after binding to STX, which was also applied in the ConCap tests. This effect was enhanced as the concentration of STX increased. As a result, a linear relationship was found between the concentrations and ConCap results, highlighting a linear detection range. As is known, aptamers have strong affinity to their targets, which was developed upon by SELEX [17]. Although the structure of PTX and OA is similar to that of STX, the interaction between toxins and aptamers is different. The strong interaction of STX with the aptamer led to conformation change in the latter, resulting in charge redistribution and an obvious potential shift. However, due to the weak interaction of PTX and OA, a potential shift was negligible.

## 5. Conclusions

In summary, a label-free aptasensor based on the Mxene-Aptamer was developed for STX detection in this study. The Mxene-Aptamer showed specific recognition capability for STX with high stability and good specificity. In the aptasensor, MXene with large surface area and rich attachment sites benefited modification of the aptamer. The conformation changes and charge redistributions after STX binding to the MXene-Aptamer led to a shift in the *C-V* curve and ConCap measurements. This aptasensor showed concentration-dependent linear responses to STX in the range of 1.0 nM to 200 nM. The detection limit of the aptasensor was 0.03 nM, comparable to complicated methods. Besides, the sensor exhibited good selectivity, which benefits its potential applications. The detection of STX in mussel tissue extraction with good recovery rate indicated the potential application of this biosensor in real sample tests. Therefore, this aptasensor provides a label-free, low-cost, and convenient approach for STX detection with high sensitivity, low detection limit, and good selectivity, indicating potential applications in many fields related to marine biological toxin detection, such as the food industry and water quality control.

## Figures and Tables

**Table 1:** Comparison of the performances of various STX aptasensors *.

Methods	Detection Limit	Incubation Time	Linear Range (nM)	References
HILIC-MS/MS	5.69 nM	30 min	27.1–754.2	42
LSPR aptasensor	8.23 nM	30 min	16.7–33,445	43
SERS aptasensor	11.7 nM	30 min	10–200	44
Electrochemical aptasensor	0.92 nM	30 min	1–400	45
Fluorescence aptasensor	10.0 nM	30 min	0–80.3	46
Electrochemical aptasensor	0.38 nM	30 min	0.9–30	47
Electrochemical aptasensor	0.03 nM	60 min	1.0–200	This work

* HILIC-MS—short for Hydrophilic Interaction Chromatography-Mass spectroscopy; LSPR—short for Localized surface plasmon resonance; SERS—short for Surface-enhanced Raman scattering.

**Table 2:** The rate recovery detection of 100 nM STX in mussel tissue extraction.

	Detection in Buffer (Potential Shifts, V)	Detection in Mussel Tissue Extraction (Potential Shifts, V)	Rate of Recovery (%)
*C_STX_* = 100 nM	0.169 ± 0.082	0.172 ± 0.025	103%
